# The Effect of Stereocomplex Polylactide Particles on the Stereocomplexation of High Molecular Weight Polylactide Blends

**DOI:** 10.3390/polym13122018

**Published:** 2021-06-21

**Authors:** Muhammad Samsuri, Ihsan Iswaldi, Purba Purnama

**Affiliations:** 1 Chemical Engineering Department, Universitas Bhayangkara Jakarta Raya, Bekasi 17121, West Java, Indonesia; msamsuri79@gmail.com; 2 School of Applied STEM, Universitas Prasetiya Mulya, Tangerang 15339, Banten, Indonesia; ihsan.iswaldi@prasetiyamulya.ac.id; 3 Vanadia Utama Science and Technology, PT Vanadia Utama, Jakarta 14470, Jakarta, Indonesia

**Keywords:** biopolymers, biodegradable polymers, mechanical properties, reinforcements, stereocomplex, polylactide, high molecular weight

## Abstract

Stereocomplexation is one of several approaches for improving polylactide (PLA) properties. The high molecular weight of poly L-lactide (PLLA) and poly D-lactide (PDLA) homopolymers are a constraint during the formation of stereocomplex PLAs (s-PLAs). The presence of s-PLA particles in PLA PLLA/PDLA blends can initiate the formation of s-PLA crystalline structures. We used the solution casting method to study the utilization of s-PLA materials from high molecular weight PLLA/PDLA blends for increasing s-PLA formation. The s-PLA particles initiated the formation of high molecular weight PLLA/PDLA blends, obtaining 49.13% s-PLA and 44.34% of the total crystalline fraction. In addition, the mechanical properties were enhanced through s-PLA crystalline formation and the increasing of total crystallinity of the PLLA/PDLA blends. The s-PLA particles supported initiation for s-PLA formation and acted as a nucleating agent for PLA homopolymers. These unique characteristics of s-PLA particles show potential to overcome the molecular weight limitation for stereocomplexation of PLLA/PDLA blends.

## 1. Introduction

Environmental concerns have driven attention toward developing degradable polymers and recycling non-degradable polymers. Replacing fossil fuel-based polymers with degradable polymers and recyclable or reusable non-degradable polymers would help mitigate environmental problems. Polylactide (PLA) is an economically feasible and bio-based polymer that has the potential to replace non-degradable polymers. PLA is biodegradable and biocompatible [1,2]. However, PLA has mechanical and thermal limitations when compared to fossil fuel-based polymers. There are many strategies to solve property limitations of PLA materials. Stereocomplex polylactide (s-PLA) is one of the methods for improving the properties of PLA.

The formation of s-PLA was discovered in an enantiomeric polymer blend of poly L-lactide (PLLA) and poly D-lactide (PDLA) with a minimum of seven units of lactide fragment [3,4]. Previous reports investigated the formation of s-PLA through solution [3,5], melt [6,7], supercritical fluid [8,9], and microwave irradiation [10]. These s-PLA formation methods can be classified in the presence of solvent [3,5,8,9,11,12,13] and in the absence of solvent [6,7,10,14,15] with their advantages and disadvantages. In the method involving solvent, the solubility of PLA homopolymer was the most important factor in obtaining large numbers of s-PLA formation. The low molecular weight PLLA and PDLA homopolymers were completely dissolved in a high-quality organic solvent, such as chloroform or dichloromethane. The higher molecular weight of the PLA homopolymers required a higher quantity of solvent to obtain proper solution and form s-PLA crystallites. The method without solvent usually required high temperatures, which were necessary for forming the molten state of the PLA homopolymer before s-PLA crystallite formation. These high temperatures are not desirable because the homopolymer can become significantly degraded [16]. The homopolymer crystal is preferentially formed during the common melt blending method for PLLA and PDLA homopolymers [17,18]. Solution casting and melt blending were the most common methods used in previous research. For both methods, the high molecular weight of PLLA and PDLA homopolymers is the main obstacle to obtain a large number of s-PLA crystallites [8,11,12,13]. Based on a previous study, blending PLLA and PDLA homopolymers with an average molecular weight (M_w_) range of approximately 125,000–150,000 obtained about 25.9% stereocomplexation degree [10]. As Tsuji and Ikada reported, the critical value of M_w_, at which only stereocomplex crystallites form, is approximately 1.0 × 10^5^ [12]. This critical value affects the solubility PLA in organic solvent, and molten state characteristic during melt process correlate to the molecular interaction during s-PLA formation.

The nucleation of s-PLA crystallites is driven by hydrogen bonding between PLLA and PDLA fragments, resulting in different crystal structures of s-PLA compared to its homopolymers [16]. Different crystal structures and nucleation characteristics produce different mechanical and thermal properties. For example, the melting temperature (T_m_) of s-PLA is approximately 50 °C higher than the T_m_ of either PLLA or PDLA [3,16]. Property improvements would allow s-PLA as a potential biodegradable material for many applications, such as nucleating agents, high-performance polymers, biodegradable fibers and films, bone plates, and dental implants [16]. Applying s-PLA materials as nucleating agents offers many benefits for PLA-based materials, especially with regard to material compatibility. In previous studies, the utilization of s-PLA particles as a nucleating agent in polymer blends improved its mechanical and thermal properties [3,16]. The presence of s-PLA crystallites increased crystallization rates and nucleation sites in PLA blends through solution [19,20] and melt process [21,22,23]. The addition of s-PLA materials into homopolymers shows the potential application of s-PLA particles to improve the crystallization rate of PLA homopolymers [19,20]. In other studies, the combination of PLLA and PDLA in melt process affects the increasing crystallization degree of PLA blends [21,22,23].

Because of their unique characteristics, s-PLA crystallites have potential as nucleating agents. They are also expected to overcome high-molecular-weight challenges in s-PLA formation from PLLA/PDLA blends. In this work, we were interested in studying the effect of s-PLA nucleation on the stereocomplex formation of PLA from high molecular weight PLLA and PDLA homopolymers through the solution casting method. The presence of s-PLA particles in high molecular weight PLLA/PDLA blends may stimulate the formation of s-PLA and also influence the change in the crystallinity degree of the blends. We evaluated the degree of s-PLA formation from PLLA/PDLA blends, assessed any improvements in mechanical properties, and studied the effect of s-PLA particle size.

## 2. Materials and Methods

### 2.1. Materials

The lactide monomers L-lactide (L-LA) and D-lactide (D-LA) were purchased from Corbion (Amsterdam, Netherlands). Tin(II)bis(2-ethylhexanoate) and stannous octoate (Sn(Oct)_2_) (Sigma Chemical Co., St. Louis, MO, USA, purity ≥ 99%) and 1-dodecanol (DoOH) (Sigma-Aldrich, purity ≥ 99.5%) were purified by distillation under reduced pressure and dissolved in dry toluene. The toluene (Sigma-Aldrich, purity ≥ 99.5%) was dried by refluxing it over a benzophenone-Na complex and distilling it in a nitrogen atmosphere immediately prior to use. Chloroform and methanol (Merck Milipore, Burlington, MA, USA, purity > 99.5%) were used as received. The s-PLA materials were synthesized from PDLA (M_n_ ≈ 87,000 g/mol; PDI = 1.437) and PLLA (M_n_ ≈ 87,000 g/mol; PDI = 1.759) blends with a 1:1 weight ratio using supercritical carbon dioxide–dichloromethane at 65 °C and 350 bar for 5 h [8]. The synthesized s-PLA materials were obtained in dry and powder-shaped form and then sieved by vibratory sieve shakers with different particle size ranges. Dry and powder-shaped s-PLA materials were classified based on particle size: <53 µm (A), 53–125 µm (B), and 125–212 µm (C).

### 2.2. PLLA and PDLA Synthesis

High molecular weight PLLA and PDLA were synthesized through ring-opening bulk polymerization of L-LA and D-LA using stannous octoate (catalyst) and 1-dodecanol (initiator) at 130 °C for 24 h. All polymerization preparation was carried out in a drying room to control humidity. The ring-opening polymerization was conducted in a round-bottom flask and completed with a magnetic stirrer and oil bath. First, the L-LA or D-LA monomers (20 g), catalyst, and initiator were added to the flask in a ratio of 1500:1:1. To remove any contaminants and moisture, the flask was purged with nitrogen gas three times and vacuumed for 6 h. The flask was then sealed under vacuum conditions and immersed in an oil bath at the desired temperature (130 °C). After polymerization was complete, the polymer products were dissolved in chloroform, precipitated in methanol, filtered, and dried in an oven at 40 °C for 24 h to obtain purified high molecular weight PLLA or PDLA.

### 2.3. Stereocomplex Formation of PLA

The stereocomplexation of PLA materials was prepared by combining the PLLA and PDLA homopolymers (1:1 weight ratio) using the solution casting method in the presence of s-PLA particles of various contents and particle sizes. We denoted samples of produced s-PLA as PLLA/PDLA_Ax, PLLA/PDLA_Bx, and PLLA/PDLA_Cx depending on their particle size category (A, B, and C, respectively). The x values represented the s-PLA content in the blends. The PLLA/PDLA_A1, PLLA/PDLA_A3, PLLA/PDLA_A5, PLLA/PDLA_A10, and PLLA/PDLA_A20 represented the PLA blends with 1%, 3%, 5%, 10%, and 20% s-PLA particle content, respectively. The original PLLA and PDLA blend was used as control material. The PLLA, PDLA, and s-PLA particle mixture was dissolved in dichloromethane with a total polymer to a total solvent ratio (weight to volume) of about 5:100. The mixture was vigorously stirred for 4 h and poured into a Petri glass. It then underwent evaporation at room temperature for 24 h and was subsequently placed in vacuum conditions at 80 °C for 48 h to obtain dry film of the polymer mixture.

### 2.4. Characterization

The polymerization conversion of PLA homopolymer was measured via gravimetric analysis. The molecular weight of the purified PLLA and PDLA homopolymers were evaluated using gel permeation chromatography (GPC; GPCmax 2001) at 40 °C, with chloroform as a solvent, a flow rate of 1.0 mL/min, and a polymer concentration of 0.2%. The monodisperse polystyrene standard was used for calibration. The synthesized PLLA/PDLA blend films were characterized to evaluate the effect of adding s-PLA materials on the degree of s-PLA formation, as well as on the mechanical and thermal properties. Melting properties were evaluated by differential scanning calorimetry (DSC) using a modulated differential scanning calorimeter (Modulated DSC 2910, TA Instrument, New Castle, UK) with a fixed heating rate of 10 °C/min, going from 0 °C to 250 °C for the homopolymer and from 0 °C to 250 °C for the PLLA/PDLA blends. The formation of s-PLA crystalline was evaluated by an X-ray diffractometer (Rigaku D/Max-2500) composed of a Cu *K_α_* source (λ = 1.54056 Ǻ, 30 kV, 100 mA), a quartz monochromator, and a goniometric plate with an observation range of 3–30° of 2*θ*.

The mechanical properties of the PLA blends were evaluated by a Universal Testing Machine (Instron 6800 Series) with an adopted method for small size samples [8]. The specimens were prepared by manual cutting of polymer mixture film from the solution casting method. Specimen size and thickness were 20 × 5 mm and 60 μm, respectively. The extension rate of the instrument was 1 mm/min, with 10 mm between each of the supports.

## 3. Results and Discussion

We expected the utilization of s-PLA crystallites to improve the stereocomplex formation of high molecular weight PLA homopolymers. In this study, we synthesized high molecular weight PLLA and PDLA homopolymers and combined them with s-PLA crystallites. We also compared the effect of s-PLA particle size on high molecular weight s-PLA formation.

**Figure 1:**
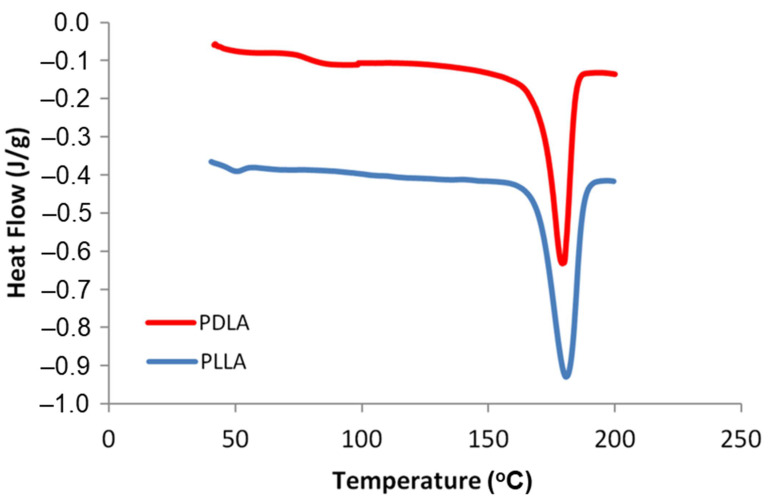
The DSC thermograms of PLLA and PDLA homopolymers produced by ring-opening bulk polymerization at 130 °C for 24 h.

We started the study by synthesis of high molecular weight PLLA and PDLA homopolymers. Ring-opening polymerization is the most well-known polymerization method that produces PLA from lactide. In this polymerization process, propagation steps were performed by the monomer molecule insert into the metal–oxygen bonds of the catalyst. Catalyst solubility is the key factor for an effective polymerization process in melt [24]. High molecular weight PLLA and PDLA were successfully synthesized through ring-opening bulk polymerization of L-LA and D-LA, with stannous octoate and 1-dodecanol as the catalyst and initiator, respectively. The high solubility of stannous octoate in molten lactide aided the polymerization process with good reproducibility [25]. The presence of synthesized PLLA and PDLA was confirmed by the melting temperature peak obtained from DSC thermograms, as shown in Figure 1.

The polymerization conversion was calculated using gravimetric analysis (see Table 1). Polymerization yields were higher than 90% due to the high solubility of stannous octoate in molten lactide. The GPC analysis of the generated PLLA and PDLA homopolymers showed a high M_w_ and a broad molecular weight distribution (MWD). High molecular weight was obtained during polymerization due to an effective ratio of the active compound, tin-alkoxide. As reported by Pack et al., the molecular weight of a PLA homopolymer is independent of the initial catalyst (stannous octoate) concentration, but it depends on the tin-alkoxide concentration formed by stannous octoate and 1-dodecanol [26]. The broad MWD may have been affected by slow initiation, chain transfer to monomers, and multiplicity of the active sites during the polymerization process [27,28].

We investigated the effect of s-PLA crystallites in the high molecular weight PLLA/PDLA blends through the solution casting method. The stereocomplex formation and the crystallinity were evaluated through DSC analysis as tabulated in Table 2. Based on our experiments, the addition of s-PLA crystallites had a significant effect on the stereocomplex formation of high molecular weight PLLA/PDLA blends. The neat high molecular weight PLLA/PDLA blend had a single peak of T_m_ around 177.85 °C. The single peak of T_m_ shows that there is no stereocomplexation between high molecular weight PLLA/PDLA. The PLLA/PDLA blend with M_w_ above 1.0 × 10^5^ restricted the formation of s-PLA crystallites [12]. High molecular weight PLLA molecules have limited chain mobility and molecular weight interaction to form s-PLA. The addition of s-PLA crystallites into high molecular weight PLLA/PDLA blends influenced the nucleation behavior of the blends by the presence of the new T_m_ above 210 °C belongs to s-PLA crystallites. The presence of s-PLA crystallites showed a significant change in the melting behavior of PLLA/PDLA blends under non-isothermal conditions. As polymer blends consist of PLA homocrystallites and s-PLA crystallites, we calculated the total melting enthalpy (∆H_m_) as follows [29]:

(1) $$\Delta H_{m}=\Delta H_{m}^{1}+\Delta H_{m}^{2}$$

where $\Delta H_{m}^{1}$ and $\Delta H_{m}^{2}$ correspond to PLA homocrystallites and s-PLA crystallites, respectively. The term ΔH_m_^0^ represents the theoretical value of the melting enthalpy of total crystal for PLA homocrystallites and s-PLA crystallites. It was calculated as follows [29]:

(2) $$\Delta H_{m}^{0}=\left(\Delta H_{m\left(\mathrm{PLA}\right)}^{0}\times \frac{\Delta H_{m}^{1}}{\Delta H_{m}^{1}+\Delta H_{m}^{2}}\right)+\left(\Delta H_{m\left(s-\mathrm{PLA}\right)}^{0}\times \frac{\Delta H_{m}^{2}}{\Delta H_{m}^{1}+\Delta H_{m}^{2}}\right)$$

This resulted in $\Delta H_{m\left(\mathrm{PLA}\right)}^{0}$ = 106 J/g for PLA homocrystallites and $\Delta H_{m\left(s-\mathrm{PLA}\right)}^{0}$ = 142 J/g for s-PLA crystallites [30]. The degree of crystallinity (X) is the ratio of total melting enthalpy to theoretical melting enthalpy for the blend [29]. The degree of stereocomplexity was calculated based on the ratio of melting enthalpy [8]:

(3) $$s-\mathrm{PLA}\;\mathrm{Degree}\;\left(\%\right)=\frac{\Delta H_{m}^{2}}{\Delta H_{m}^{1}+\Delta H_{m}^{2}}\times 100$$

Based on experimental data, the addition of s-PLA crystallites increased the total crystallinity of the PLLA/PDLA blends concurrently with the degree of s-PLA formation, as shown in Table 2. The increasing of s-PLA crystallites content increased the total crystallinity and degree of s-PLA up to a certain level, then decreased. The PLLA/PDLA_A10 sample had a slightly higher total crystallinity value but a lower level of s-PLA compared to PLLA/PDLA_A5. The total crystallinity of PLLA/PDLA_B20 was drastically decreased even though it contained 32.77% of s-PLA degree. The high content of s-PLA crystallites may form aggregates, which counter the nucleation effect. It aligned with previous research where the addition of s-PLA crystallites improved polymer crystallinity [19,20]. For particle size effect, the PLLA/PDLA_B10 and PLLA/PDLA_C10 samples showed higher levels of s-PLA but lower total crystallinity values compared to PLLA/PDLA_A10. The bigger particle size decreased the number of nucleating sites, which contributed to the increased levels of s-PLA. Based on this, the s-PLA crystallites acted as an initiator for s-PLA formation and as a nucleating agent for PLA homopolymers. The s-PLA markedly promoted the formation of s-PLA from high molecular weight PLLA/PDLA blends.

**Figure 2:**
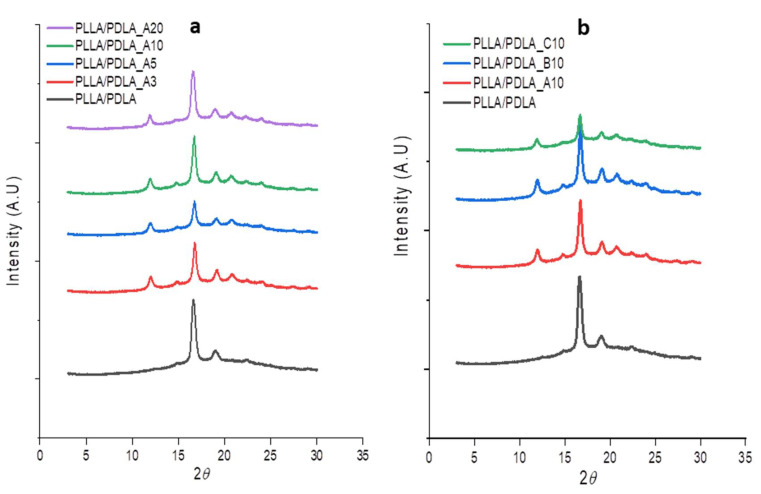
The XRD pattern of PLLA/PDLA blends: (**a**) PLLA/PDLA blends with different s-PLA crystallites contents, (**b**) PLLA/PDLA blends with different s-PLA particle sizes.

The DSC data for s-PLA formation from high molecular weight PLLA/PDLA blends were also confirmed with XRD results, as shown in Figure 2. From XRD data, the crystalline structure of s-PLA showed different characteristic peaks compared to PLA homocrystallines. The left- and right-handed helical conformations of PLLA and PDLA were packed in parallel fashion and formed a new crystalline s-PLA structure [31]. PLLA/PDLA homopolymers showed clear diffraction peaks at 17° and 19°, while the PLLA/PDLA blends containing s-PLA crystallites showed additional characteristic peaks at 12° and 21°, as well as small peaks at 24°. The presence of s-PLA characteristic peak indicates that s-PLA crystallites act as an initiator for s-PLA formation in high molecular weight PLLA/PDLA blends. As reported in a previous study, the presence of s-PLA crystallites also initiates a nucleation site of PLA blends [20]. The possible mechanism may start with the initiating of s-PLA formation up to a certain portion, followed by the nucleation of homopolymers.

**Figure 3:**
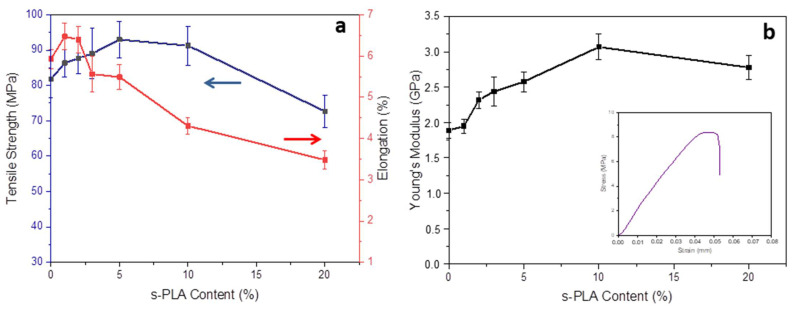
The mechanical properties of PLLA/PDLA blend with various s-PLA contents (**a**) tensile strength and elongation (%), (**b**) Young’s modulus (insert: Typical stress-strain curve).

The increasing total crystallinity and s-PLA degree of PLLA/PDLA blends affect the change in their mechanical properties. The mechanical properties of PLLA/PDLA blends are shown in Figure 3. As the highest s-PLA degree was obtained in PLLA/PDLA_A5, the highest tensile strength was also obtained at 5% s-PLA content. Young’s modulus increased up to 10% of s-PLA content then decreased at a higher content. With increasing s-PLA degree, the elongation at break started to decrease at 3% of s-PLA content. As increasing s-PLA content influenced the increasing s-PLA degree and total crystallinity, it will enhance mechanical properties, but it will also affect to the decreasing elongation at break. The particle size of s-PLA crystalline also affected the mechanical properties of the PLLA/PDLA blends (see Table 3). Smaller particles more significantly improved tensile strength, Young’s modulus, and elongation at break. Smaller particles also contributed to the higher number of initiation sites for s-PLA formation and of nucleation sites for PLA homopolymers.

## 4. Conclusions

The stereocomplexation of PLA is one of the more suitable approaches for improving PLA properties. The s-PLA formation from PLLA/PDLA blends was restricted by molecular weight constraints. The presence of s-PLA materials in the high molecular weight PLLA/PDLA homopolymers can initiate the s-PLA formation. The addition of 5% s-PLA particles in high molecular weight PLLA/PDLA blends triggered s-PLA formation up to 49.13% s-PLA degree and 44.34% of the total crystalline fraction. At 10% s-PLA, the formation of s-PLA decreased to 42.75%, but the total crystalline fraction reached 45.07%. The s-PLA particles have capabilities to initiate s-PLA formation and also act as a nucleating agent for the PLA homopolymers. The higher s-PLA degrees and crystalline fraction in the PLLA/PDLA blends aligned with the mechanical property improvements in the PLLA/PDLA blends. The particle size of the s-PLA materials influenced s-PLA formation, total crystalline fraction, and the mechanical properties of the PLLA/PDLA blends. Smaller particle sizes contributed to better particle distribution, as well as more initiation sites for s-PLA formation and nucleation sites for the PLA homopolymer. The s-PLA particles showed the capability to overcome the molecular weight limitation in PLA stereocomplexation.

## Figures and Tables

**Table 1:** PLA homopolymer synthesis through bulk ring-opening polymerization of lactide at 130 °C with reaction time 24 h.

Materials	Yield (%)	Mn	Mw	MWD
PLLA	95.16 ± 3.84	195,322	395,141	2.02
PDLA	94.40 ± 4.82	170,035	459,042	2.70

**Table 2:** Thermal evaluation of s-PLA formation through solution casting with various s-PLA crystallites content by DSC instrument.

Materials	T_m_^1^ (°C)	∆H_m_^1^ (J/g)	T_m_^2^ (°C)	∆H_m_^2^ (J/g)	∆H_m_ (J/g)	∆H_m_^0^ (J/g)	X (%)	s-PLA Degree (%)
PLLA/PDLA	177.85	39.85	-	-	39.89	106.00	37.59	0
PLLA/PDLA_A1	177.48	32.87	226.73	15.05	47.92	117.31	40.85	31.41
PLLA/PDLA_A3	179.87	27.86	226.39	22.63	50.49	122.14	41.34	44.83
PLLA/PDLA_A5	180,26	27.80	219.28	26.85	54.66	123.69	44.34	49.13
PLLA/PDLA_A10	179.47	31.32	221.38	23.39	54.71	121.39	45.07	42.75
PLLA/PDLA_A20	176.98	26.92	224.47	13.12	40.04	117.80	34.00	32.77
PLLA/PDLA_B10	178.50	28.08	221.99	23.06	51.14	122.23	41.84	45.09
PLLA/PDLA_C10	179.67	26.88	223.43	25.30	52.19	123.46	42.27	48.49

**Table 3:** The mechanical properties of PLLA/PDLA blends with different particle sizes of s-PLA.

Materials	Elongation at Break (%)	Tensile Strength (MPa)	Young’s Modulus (GPa)
PLLA/PDLA	5.90 ± 0.23	81.85 ± 5.32	1.89 ± 0.11
PLLA/PDLA_A10	4.30 ± 0.20	91.27 ± 5.48	3.07 ± 0.18
PLLA/PDLA_B10	4.22 ± 0.22	83.84 ± 3.02	2.76 ± 0.13
PLLA/PDLA_C10	4.12 ± 0.23	64.95 ± 4.29	2.50 ± 0.14

## Data Availability

Data is contained within the article.
